# Assimilation of arabinogalactan side chains with novel 3-*O*-β-L-arabinopyranosyl-α-L-arabinofuranosidase in *Bifidobacterium pseudocatenulatum*

**DOI:** 10.20517/mrr.2023.08

**Published:** 2023-04-15

**Authors:** Yuki Sasaki, Makoto Yanagita, Mimika Hashiguchi, Ayako Horigome, Jin-Zhong Xiao, Toshitaka Odamaki, Kanefumi Kitahara, Kiyotaka Fujita

**Affiliations:** ^1^Faculty of Agriculture, Kagoshima University, Kagoshima, Kagoshima 890-0065, Japan.; ^2^Graduate School of Biostudies, Kyoto University, Sakyo-ku, Kyoto 606-8502, Japan.; ^3^Next Generation Science Institute, Morinaga Milk Industry Co. Ltd., Zama, Kanagawa 252-8583, Japan.

**Keywords:** *Bifidobacterium pseudocatenulatum*, arabinogalactan protein, type II arabinogalactan, glycoside hydrolase

## Abstract

**Aim:** Dietary plant fibers affect gut microbiota composition; however, the underlying microbial degradation pathways are not fully understood. We previously discovered 3-*O*-α-D-galactosyl-α-L-arabinofuranosidase (GAfase), a glycoside hydrolase family 39 enzyme involved in the assimilation of side chains of arabinogalactan protein (AGP), from *Bifidobacterium longum * subsp. *longum* (*B. longum*) JCM7052. Although GAfase homologs are not highly prevalent in the *Bifidobacterium* genus, several *Bifidobacterium* strains possess the homologs. To explore the differences in substrate specificity among the homologs, a homolog of *B. longum* GAfase in *Bifidobacterium pseudocatenulatum* MCC10289 (MCC10289_0425) was characterized.

**Methods:** Gum arabic, larch, wheat AGP, and sugar beet arabinan were used to determine the substrate specificity of the MCC10289_0425 protein. An amino acid replacement was introduced into GAfase to identify a critical residue that governs the differentiation of substrate specificity. The growth of several *Bifidobacterium* strains on β-L-arabinopyranosyl disaccharide and larch AGP was examined.

**Results:** MCC10289_0425 was identified to be an unprecedented 3-*O*-β-L-arabinopyranosyl-α-L-arabinofuranosidase (AAfase) with low GAfase activity. A single amino acid replacement (Asn^119^ to Tyr) at the catalytic site converted GAfase into AAfase. AAfase releases sugar source from AGP, thereby allowing *B. pseudocatenulatum* growth.

**Conclusion:** Bifidobacteria have evolved several homologous enzymes with overlapping but distinct substrate specificities depending on the species. They have acquired different fitness abilities to respond to diverse plant polysaccharide structures.

## INTRODUCTION

Bifidobacteria are symbionts in the human gut and produce several carbohydrate-hydrolyzing enzymes to break down sugars. To control the presence and proliferation of beneficial bacteria in the intestinal tract, it is essential to understand the degradation and metabolic basis for individual sugar sources, such as milk oligosaccharides and dietary fibers. Bifidobacteria generally utilize oligosaccharides with relatively low molecular weight as an energy source and receive degradative products from other symbiont bacteria, such as *Bacteroides*^[1]^. However, several *Bifidobacterium* species, particularly the adult type (*Bifidobacterium longum* subsp. *longum*, *B. adolescentis*, *B. pseudocatenulatum*, and *B. catenulatum*), possess extracellular carbohydrate-hydrolyzing enzymes that can access polysaccharides and glycoproteins directly, thereby enabling the production of transportable small saccharides from those such as resistant starch^[2]^, arabinan^[3]^, mannan^[4]^, extensin^[5,6]^, arabinogalactan protein (AGP)^[7-9]^, and arabinoxylan^[10]^.

The structure of AGP is common in higher plant cell walls. In particular, the structures of type II arabinogalactan (AG) moieties from gum arabic and larch wood have been well studied and comprise a β-1,3-galactan backbone and β-1,6-galactan side chains with several modifications of other sugars^[11-13]^. Recently, we elucidated the molecular basis of assimilating gum arabic AGP in *B. longum* JCM7052^[8,9]^. The extracellular glycoside hydrolase (GH) family 39 enzyme "3-*O*-α-D-galactosyl-α-L-arabinofuranosidase (GAfase)" can act on gum arabic AGP and facilitate the action of other enzymes for degrading the AGP backbone and modified sugar. Gum arabic AGP consists of α-D-Gal-(1→3)-α-L-Ara*f*-(1→3) and β-L-Ara*p*-(1→3)-α-L-Ara*f*-(1→3) structures, and larch AGP consists of β-L-Ara*p*-(1→3)-α-L-Ara*f*-(1→3) structure in the side chain. GAfase weakly cleaves β-L-Ara*p*-(1→3)-α-L-Ara*f*-(1→3) linkage in addition to α-D-Gal-(1→3)-α-L-Ara*f*-(1→3) linkage due to their structural similarity. A homology search revealed that GAfase homolog genes are found across other *Bifidobacterium* species. Moreover, based on sequence identity and peripheral genetic composition, the activities of these homologous enzymes were predicted to be different from GAfase.

In this study, we performed a functional analysis of *B. pseudocatenulatum* MCC10289_0425, which has 60% amino acid sequence identity to GAfase, a novel 3-*O*-β-L-arabinopyranosyl-α-L-arabinofuranosidase (AAfase). AAfase preferentially releases β-L-Ara*p*-(1→3)-L-Ara over α-D-Gal-(1→3)-L-Ara; although its function overlaps with that of GAfase from *B. longum*, both enzymes have distinct substrate specificities. Furthermore, a mutagenesis study revealed the critical amino acid that governs the differentiation of substrate specificity between AAfase and GAfase. We discussed an example demonstrating diversified specificity in GH39 within the *Bifidobacterium* genus. Bifidobacteria might have acquired different fitness abilities to respond to diverse plant polysaccharide structures.

## METHODS

### Materials

Gum arabic AGP was obtained from Sigma-Aldrich (St. Louis, MO, USA). Larch AGP was purchased from Tokyo Chemical Industry Co., Ltd. (Tokyo, Japan). Sugar beet arabinan was obtained from Megazyme (Wicklow, Ireland). The polysaccharides were purified via ethanol precipitation of the aqueous solutions to remove releasing sugars with low molecular weight from the original reagent. Wheat AGP was purified according to the method described by Fincher *et al*. with some modifications^[14]^. Briefly, wheat flour was dried and heated to remove endogenous enzymes, and extraction was performed with 80% ethanol under reflux for 30 min. Ethanol-insoluble residues were collected on a glass fiber filter and resolved in water at 37 °C for 60 min. After centrifugation, the water-soluble fraction was digested with α-amylase and amyloglucosidase to remove starch. After dialysis, the solution was mixed gradually with ethanol until a final concentration of 65% to separate arabinoxylan and AGP. The supernatant containing AGP was collected, and ethanol was added to achieve a final concentration of 80%. The precipitate was collected via glass fiber filtration, washed several times, and resolved in water. The oligosaccharides (S3-GA, S3-AA, and S5-GA) were obtained from the supernatant following the ethanol precipitation of gum arabic reagent^[8]^, based on the method described by Tischer *et al*.^[15]^ β-L-Ara*p*-(1→3)-α-L-Ara*f*-OMe and α-D-Gal*p*-(1→3)-α-L-Ara*f*-OMe were prepared via the transglycosylation of GAfase with gum arabic and larch AGP, respectively^[8]^. pET23d_GAfase plasmid and recombinant GAfase were prepared as described previously^[8]^.

### Expression and purification of recombinant AAfase

The sequence of MCC10289_0425 was obtained from the genome of *B. pseudocatenulatum* MCC10289 (F03Father01; accession number: SAMN09671256)^[16]^. The codon-optimized sequence of MCC10289_0425 without the N-terminal signal peptide (SP; aa 1-28) and with C-terminal His-tag and N-terminal SKIK-tag was synthesized and cloned into a pET23a vector via GenScript (Nanjing, China). The synthesized pET23a-MCC10289_0425 plasmid was transformed into *Escherichia coli* BL21 (DE3) pLysS cells (BioDynamics Laboratory, Tokyo, Japan), which were cultured at 37 °C for 3 h. The cells were induced with 1 mM IPTG at 15 °C for 44 h. The cultured cells were centrifuged, and the resultant pellet was resuspended in xTractor™ buffer. The target protein was purified using Capturem™ His-Tagged Purification Maxiprep columns (Takara Bio Inc., Shiga, Japan) according to the manufacturer’s instructions. Desalination and concentration of the target protein were performed using ultrafiltration membranes [molecular-weight cutoff (MWCO), 10 kDa; Millipore Co., Billerica, MA, USA].

### Enzyme assay

The reactivity of the recombinant enzyme was analyzed using the following polysaccharide substrates: gum arabic, larch, wheat AGP, and sugar beet arabinan. The hydrolytic activity of AAfase was evaluated using polysaccharides (1.0%) in 40 µL of 50 mM sodium acetate buffer (pH 5.5). After incubating the mixture at 37 °C for 16 h, the reaction products were analyzed via thin layer chromatography (TLC) and high-performance anion-exchange chromatography with pulsed amperometric detection (HPAEC-PAD). For TLC analysis, the reaction products were spotted on a silica gel 60 aluminum plate (Merck, Darmstadt, Germany) with an *n*-propanol/ethanol/water mixture [7:1:2 (v/v/v)]. Sugars were visualized by spraying the orcinol-sulfate reagent on the plates^[17]^. For HPAEC-PAD, the reaction products were analyzed via CarboPac PA-1 columns (φ, 4 mm × 250 mm; Dionex Corp., Sunnyvale, CA, USA) with a flow rate of 1.0 mL/min using the following gradient: 0-5 min, 100% eluent A (0.1 M NaOH); 5-30 min, 0%-100% eluent B (0.5 M sodium acetate and 0.1 M NaOH); and 30-35 min, 100% eluent B.

The hydrolytic activity against oligosaccharides (S3-AA, S3-GA, and S5-GA) was analyzed as follows. Briefly, oligosaccharides (final concentration: 1.25 µM) were incubated with 0.05 µg/mL AAfase in 50 mM MES buffer (pH 6.5) or 0.05 µg/mL GAfase in 50 mM sodium acetate buffer (pH 4.5) at 37 °C for 24 h. The reaction products were analyzed via HPAEC-PAD with CarboPac PA-1 columns (φ, 2 mm × 250 mm; Dionex Corp., Sunnyvale, CA, USA) with a flow rate of 0.25 mL/min using the following gradient: 0-5 min, 40% eluent A (0.25 M NaOH), 0.5% eluent B (1 M sodium acetate), and 59.5% eluent C (ultrapure water); 5-30 min, 40% eluent A, 0.5%-50% eluent B, and 59.5%-10% eluent C; and 30-35 min, 40% eluent A, 50% eluent B, and 10% eluent C.

### Kinetic analysis

The AAfase kinetic parameter was determined using 0.313-20.0 mg/mL larch AGP as a substrate. Larch AGP was incubated with 35.3 ng/mL AAfase in 50 mM sodium acetate buffer (pH 6.0) at 45 °C for 20 min. The reactions were terminated by adding 5% trichloroacetic acid (TCA) to one-fifth of the reaction mixture. The products were analyzed via HPAEC-PAD using CarboPac PA-1 columns (φ, 4 mm × 250 mm). The concentrations of the reaction products were calculated based on the peak areas.

### Optimal pH and temperature

The optimal pH for enzyme activity was determined by using larch AGP as a substrate in 50 mM sodium acetate (pH 3.5-6.0), MES (pH 5.5-7.0), and HEPES (pH 7.0-8.0) buffers at 45 °C. The optimal temperature for enzyme activity was determined using 50 mM sodium acetate buffer (pH 6.5) at 25 °C-60 °C. The samples were preincubated at each temperature for 5 min and were then incubated with the enzyme for 20 min at each temperature. The reactions were stopped by boiling the samples for 3 min, and the BCA reagent was used to measure the content of reducing sugar^[18]^.

### Site-directed mutagenesis

A KOD Plus mutagenesis kit (Toyobo Co., Ltd., Osaka, Japan) was used to introduce N119Y and N119D amino acid substitutions into pET23d_GAfase using specific primers [Table 1]. pET23d_GAfase_N119Y and pET23d_GAfase_N119D mutants were cloned into *E. coli* BL21 (λDE3) cells (Nippon Gene, Toyama, Japan), which were cultured at 37 °C for 2 h followed by 25 °C for 17 h using the Overnight Express autoinduction system (Novagen Inc., Wisconsin, USA). The cultured cells were centrifuged, and the resultant pellet was resuspended in BugBuster protein-extraction reagent (Novagen). The C-terminal His-tagged GAfase N119Y protein was purified using a column containing Talon metal-affinity resin (Clontech, CA, USA). Then, 25 and 50 mM imidazole fractions containing GAfase N119Y were desalted and concentrated using an ultrafiltration membrane (MWCO, 10 kDa; Millipore Co., Billerica, MA, USA). Since the activity of GAfase N119D was lost after desalting, we used a predesalted fraction containing imidazole to assess GAfase N119D activity after the resuspended pellet was purified via Capturem™ His-Tagged Purification Miniprep columns (Takara) according to the manufacturer’s instructions.

**Table 1:** Primers for site-directed mutagenesis

	**Sequence**
GAfase_mut_for	5′-CTTCAGGATTGGTACCCGGA-3’
N119Ymut_rev	5′-ATAGACCACCAGCTCCTCG-3’
N119Dmut_rev	5′-ATCGACCACCAGCTCCTCG-3’

### Construction of structural models using AlphaFold2 

The structural models of AAfase and GAfase were predicted based on the protein sequence using AlphaFold2 (ColabFold) with default settings (https://alphafold.ebi.ac.uk/)^[19,20]^. Visualization was performed using PyMOL 2.5.0 (Schrödinger LLC, NY, USA).

### Substrate specificity of AAfase, GAfase, and GAfase N119Y mutants

α-D-Gal*p*-(1→3)-α-L-Ara*f*-OMe and β-L-Ara*p*-(1→3)-α-L-Ara*f*-OMe (final concentration, 0.5 mM) were incubated with the enzymes at appropriate concentrations at 45 °C. A 50 mM MES buffer (pH 6.5) was used for AAfase and 50 mM sodium acetate buffer (pH 4.5) was used for GAfase and GAfase_N119Y. α-D-Gal*p*-(1→3)-α-L-Ara*f*-OMe was incubated with AAfase (final concentration, 12.5 µg/mL) for 90 min and GAfase (final concentration, 0.125 µg/mL) or GAfase_N119Y (final concentration, 12.5 µg/mL) for 20 min. β-L-Ara*p*-(1→3)-α-L-Ara*f*-OMe was incubated with AAfase (final concentration, 0.125 µg/mL) or GAfase_N119Y (final concentration, 0.625 µg/mL) for 20 min and GAfase (final concentration, 12.5 µg/mL) for 90 min. The reaction was terminated by adding 5% TCA to one-fifth of the mixture. The diluted products were analyzed via HPAEC-PAD using CarboPac PA-1 columns (φ, 4 mm × 250 mm), as described above. One unit of enzyme activity was defined as the amount of enzyme required to produce 1 μmol α-D-Gal*p*-(1→3)-L-Ara or β-L-Ara*p*-(1→3)-L-Ara per min.

### *In vitro* assimilation test of β-L-Ara*p*-(1→3)-L-Ara and larch AGP using *Bifidobacterium*

The three strains of *B. pseudocatenulatum* (MCC10289, MCC10285, and MCC10311) and one strain of *B. kashiwanohense* (MCC10250) used in the assimilation tests of β-L-Ara*p*-(1→3)-L-Ara and larch AGP were obtained from the stock cultures maintained at the Morinaga Milk Industry Co., Ltd., Zama, Japan. The genome sequences have been submitted to GenBank^[16]^ and the accession number are as follows: *B. pseudocatenulatum* MCC10285 (F02Son03), SAMN09671251; *B. pseudocatenulatum* MCC10311 (F04Father01), SAMN09671277; and *B. kashiwanohense* MCC10250 (F01Mother01), SAMN09671224. *B. pseudocatenulatum* JCM1200^T^ was obtained from the Japan Collection of Microorganisms (RIKEN Bioresource Center, Ibaraki, Japan). The strains were precultured at 37 °C under anaerobic conditions in MRS broth containing 0.05% L-cysteine hydrochloride (MRS + Cys) using an AnaeroPack system (Mitsubishi Gas Chemical, Tokyo, Japan). The precultured cells were inoculated into the MRS + Cys medium containing 1% L-arabinose and 1% larch AGP and incubated at 37 °C under anaerobic conditions until all strains showed adequate growth to induce gene expression prior to primary cultivation. Subsequently, the precultured cells were inoculated into the MRS + Cys medium containing 0.2% β-L-Ara*p*-(1→3)-L-Ara as a sole carbon source and were cultured for 48 h at 37 °C under anaerobic conditions. The absorbance of each culture medium was measured at time points of 17, 24, 41, and 48 h and a wavelength of 600 nm. The experiment was performed only once because of the insufficient sugar source. For the assimilation test of larch AGP, bacterial cells were precultured (as described above), inoculated into MRS + Cys medium containing 5% larch AGP, and cultured for 48 h at 37 °C under anaerobic conditions. Absorbance was measured at time points of 18, 24, 42, and 48 h and a wavelength of 600 nm. The experiment was performed in triplicates. To analyze residual sugar after cultivation, the supernatant of the culture medium of β-L-Ara*p*-(1→3)-L-Ara and larch AGP after 41 and 48 h, respectively, were analyzed through TLC and HPAEC-PAD using CarboPac PA-1 columns (φ, 4 mm × 250 mm), as mentioned above. To assess the enzymatic activity of bacterial cells and the supernatant of culture medium, pellets and supernatant of all strains cultured in MRS + Cys medium containing larch AGP as a sole carbon source for 18 h were collected and incubated with larch AGP in 50 mM sodium acetate buffer (pH 6.0) for 22 h. The reaction products were analyzed via TLC, as described above.

## RESULTS

### Gene cluster including GH39 and GH36 in *B. pseudocatenulatum*

**Figure 1:**
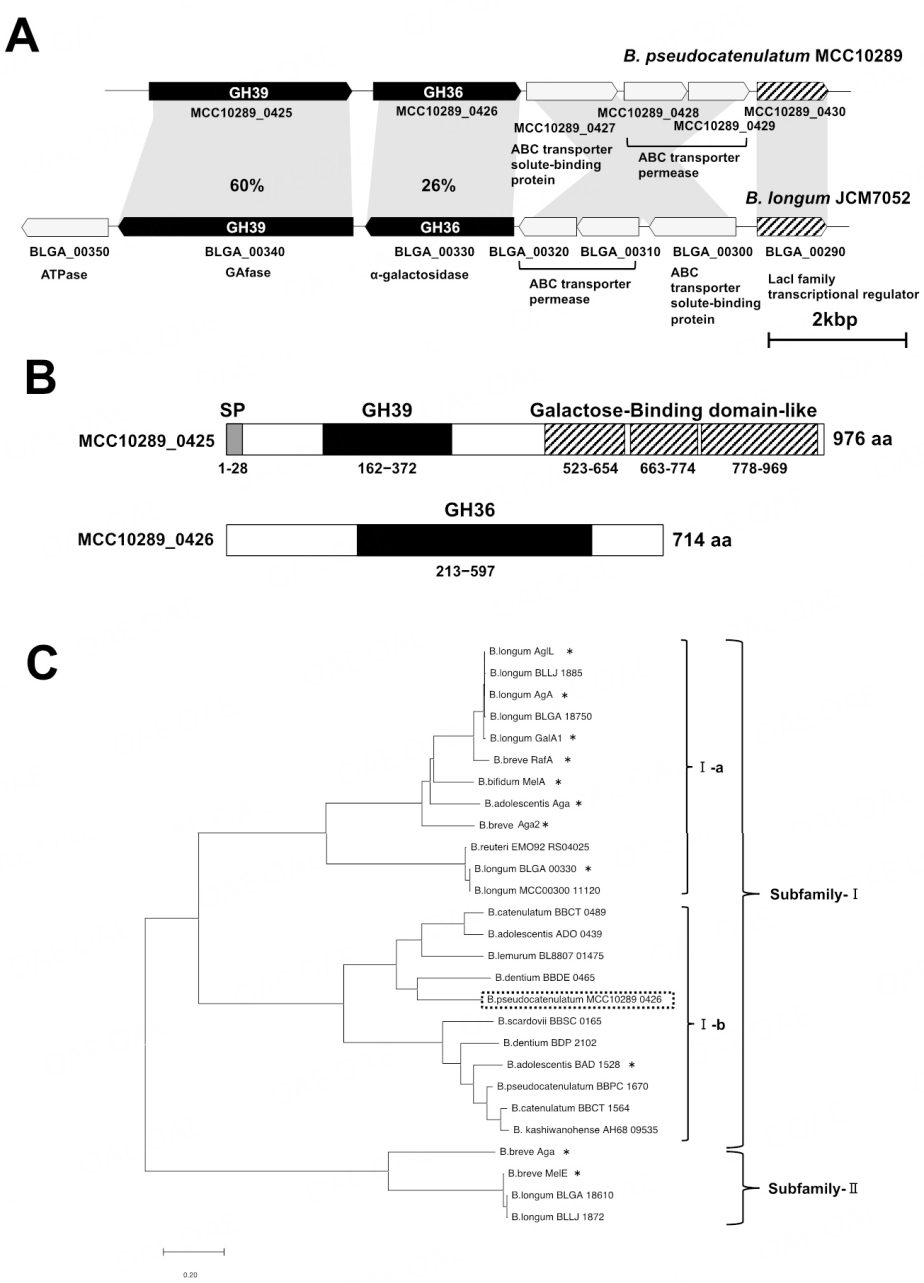
Glycoside hydrolases involved in the degradation of AGP side chains. A: Gene clusters in *Bifidobacterium pseudocatenulatum* MCC10289 and *B. longum* JCM7052 involved in the degradation of AGP. The glycoside hydrolases (GHs) in the Carbohydrate-Active EnZymes (CAZy) Database are shown inside the black arrowheads. The shaded arrows indicate the transcriptional regulator and the light gray arrows indicate the ABC transporter. The percentage in the light gray bars indicates sequence identity between GHs from *B. pseudocatenulatum* and *B. longum*; B: The domain structure of MCC10289_0425 and MCC10289_0426 in *B. pseudocatenulatum* MCC10289. The domain structures were predicted using SignalP5.0 (http://www.cbs.dtu.dk/services/SignalP/) and InterPro (https://www.ebi.ac.uk/interpro/) servers. The structure comprises the SP, GH39 catalytic domain, and three galactose-binding domain-like regions; C: The phylogenetic relationships of bifidobacterial GH36 α-galactosidases and β-L-arabinopyranosidases. The phylogenetic tree of *B. pseudocatenulatum* MCC10289_0426 with homologous proteins from bifidobacteria was constructed by the neighbor-joining method using the aligned sequences; for the construction, the program Clustal W was implemented in the MEGA11 software. *B. pseudocatenulatum*_MCC10289_0426 is indicated by a dashed-line box. The protein names or locus tags are shown alongside *Bifidobacterium* strains as follows: *B. adolescentis* Aga (GenBank ID: AAD30994.2), *B. bifidum* MelA (ABD96085.1), *B. breve* Aga (AAK96217.2), *B. breve* Aga2 (ABB76662.1), *B. longum* GalA1 (ACD98928.1), *B. longum* AgA (AAN25312.1), *B. longum* AglL (AAG02023.1), *B. breve* RafA (ABE96531.1), *B. breve* MelE (ABE96518.1), *B. longum* BLLJ_1872 (BAJ67536.1), *B. longum* BLLJ_1885 (BAJ67549.1), *B. longum* BlAga3 (BBV22622.1), *B. longum* BlAga1 (BBV24464.1), and *B. longum* BlAga2 (BBV24450.1), *B. longum* MCC00300_11120 (WP_077381863.1), *B. reuteri* EMO92_RS04025 (WP_150335335.1), *B. catenulatum* BBCT_0489 (BAR01457.1 ), *B. adolescentis* BADO_0439 (AII75864.1), *B. lemurum* BL8807_01475 (QOL34630.1), *B. dentium* BBDE_0465 (BAQ26459.1), *B. adolescentis* BAD_1528 (BBD14080.1), *B. pseudocatenulatum* BBPC_1670 (BAR04348.1), *B. catenulatum* BBCT_1564 (BAR02532.1), *B. kashiwanohense* AH68_09535 (AIZ15229.1), *B. dentium* BDP_2102 (ADB10663.1), and *B. scardovii* BBSC_0165 (BAQ30245.1). Asterisk indicates characterized enzymes.

We previously characterized GH39 GAfase and GH36 α-galactosidase in the gene cluster for utilizing gum arabic AGP in *B. longum* JCM7052^[8,21]^. A homology search of GAfase revealed that GH39 candidates were conserved in other *Bifidobacterium* species. Based on sequence identity and peripheral genetic composition, a GH39 candidate in *B. pseudocatenulatum* was expected to exhibit a different function from GAfase. In this study, we focused on a gene cluster in *B. pseudocatenulatum* MCC10289 containing the GH39 GAfase homolog MCC10289_0425 named “3-*O*-β-L-arabinopyranosyl-α-L-arabinofuranosidase (AAfase)” (Accession no. LC745705). This gene cluster was similar to that in *B. longum* JCM7052 and comprised conserved neighboring putative GH36 enzyme (MCC10289_0426), Lac-I-type transcriptional regulator (MCC10289_0430), extracellular solute-binding protein (MCC10289_0427), and ABC transporter permeases (MCC10289_0428 and MCC10289_0429) [Figure 1A]. In contrast, the homologous gene encoding putative ATPase in *B. longum* JCM 7052 (BLGA_00350) was not found in the gene cluster of *B. pseudocatenulatum*. AAfase (MCC10289_0425) and putative β-L-arabinopyranosidase (MCC10289_0426) exhibited 60% and 26% amino acid sequence identities with GAfase (BLGA_00340) and GH36 α-galactosidase (BLGA_00330, BlAga3), respectively. AAfase contains a putative SP, GH39 catalytic domain, and three galactose-binding domains according to SignalP and InterPro [Figure 1B]. GAfase is a bacterial cell wall anchoring protein, while AAfase has no transmembrane region, suggesting that it is a secreted enzyme. Although most of the *Bifidobacterium* GH36s reported so far have been characterized as α-galactosidases, BAD_1528 from *Bifidobacterium adolescentis* ATCC 15703 was the first GH36 member to be characterized as a β-L-arabinopyranosidase^[22]^. A phylogenetic tree of MCC10289_0426 was constructed using several sequences belonging to the GH36 family [Figure 1C]. The GH36 family is classified into four subfamilies^[23]^, and the enzymes characterized in bifidobacteria mainly belong to subfamilies-I and -II. The phylogenetic tree showed that the GH36 subfamily-I can be further divided into a group with α-galactosidase activity, including many characterized α-galactosidases (Subfamily-I-a group) and a group with possible β-L-arabinopyranosidase activity, including BAD_1528 (Subfamily-I-b group). Although MCC10289_0426 failed to express as a soluble protein in *E. coli*, our findings indicate that BBCT_0489 in the Subfamily-I-b group is a β-L-arabinopyranosidase (unpublished data). Therefore, we predict that MCC10289_0426 of the Subfamily-I-b group is a β-L-arabinopyranosidase and not an α-galactosidase; moreover, the neighboring GH39 GAfase homolog was predicted to release β-L-Ara*p*-(1→3)-L-Ara rather than α-D-Gal-(1→3)-L-Ara.

Recombinant AAfase was designed without the N-terminal SP (aa 1-28) and with C-terminal His-tag and N-terminal SKIK-tag. *E. coli* BL21 (DE3) plysS cells transformed with pET23a–MCC10289_0425 were cultured at 37 °C for 3 h, induced with IPTG, and cultured at 15 °C for 44 h. The protein was purified using the C-terminal His-tag. Purified recombinant AAfase migrated as a single band on SDS-PAGE, with an apparent molecular mass of 104 kDa, corresponding to its calculated molecular mass of 104,092 Da [Supplementary Figure 1]. 

### Substrate specificity and general properties of AAfase

**Figure 2:**
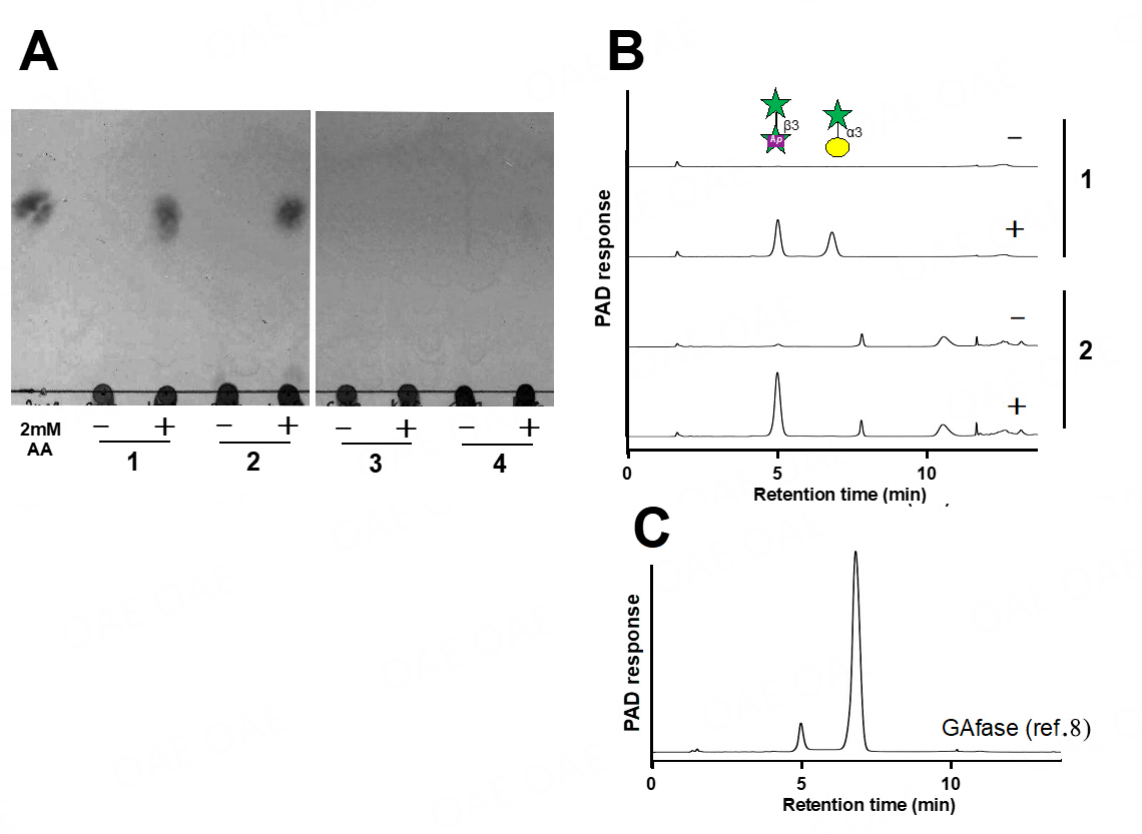
AAfase reaction with polysaccharides. TLC (A) and HPAEC-PAD (B) analyses of AAfase reaction with 1% gum arabic AGP (lane 1), larch AGP (lane 2), wheat AGP (lane 3), and sugar beet arabinan (lane 4). The polysaccharides were incubated in the absence (-) or presence (+) of AAfase. HPAEC-PAD analysis of the reaction mixture of wheat AGP and sugar beet arabinan is not shown. (C) The HPAEC-PAD data for GAfase reaction with gum arabic AGP had been published previously^[8]^. AA indicates β-L-Ara*p*-(1→3)-L-Ara.

We determined the ability of gum arabic, larch, wheat AGP, and sugar beet arabinan to serve as potential substrates for AAfase [Figure 2]. TLC and HPAEC-PAD analyses revealed that AAfase released β-L-Ara*p*-(1→3)-L-Ara and α-D-Gal*p*-(1→3)-L-Ara from gum arabic AGP and β-L-Ara*p*-(1→3)-L-Ara from larch AGP. AAfase cleaved a small amount of β-L-Ara*p*-(1→3)-L-Ara from sugar beet arabinan, but wheat AGP was not utilized as a substrate. The reaction products were identical to those cleaved by GAfase, the chemical structures of which had already been determined via enzymatic and NMR analyses in our previous study^[8]^. However, HPAEC-PAD analysis showed that the amount of α-D-Gal*p*-(1→3)-L-Ara released from gum arabic AGP by AAfase was not equivalent to that released by GAfase [Figure 2C]. 

**Figure 3:**
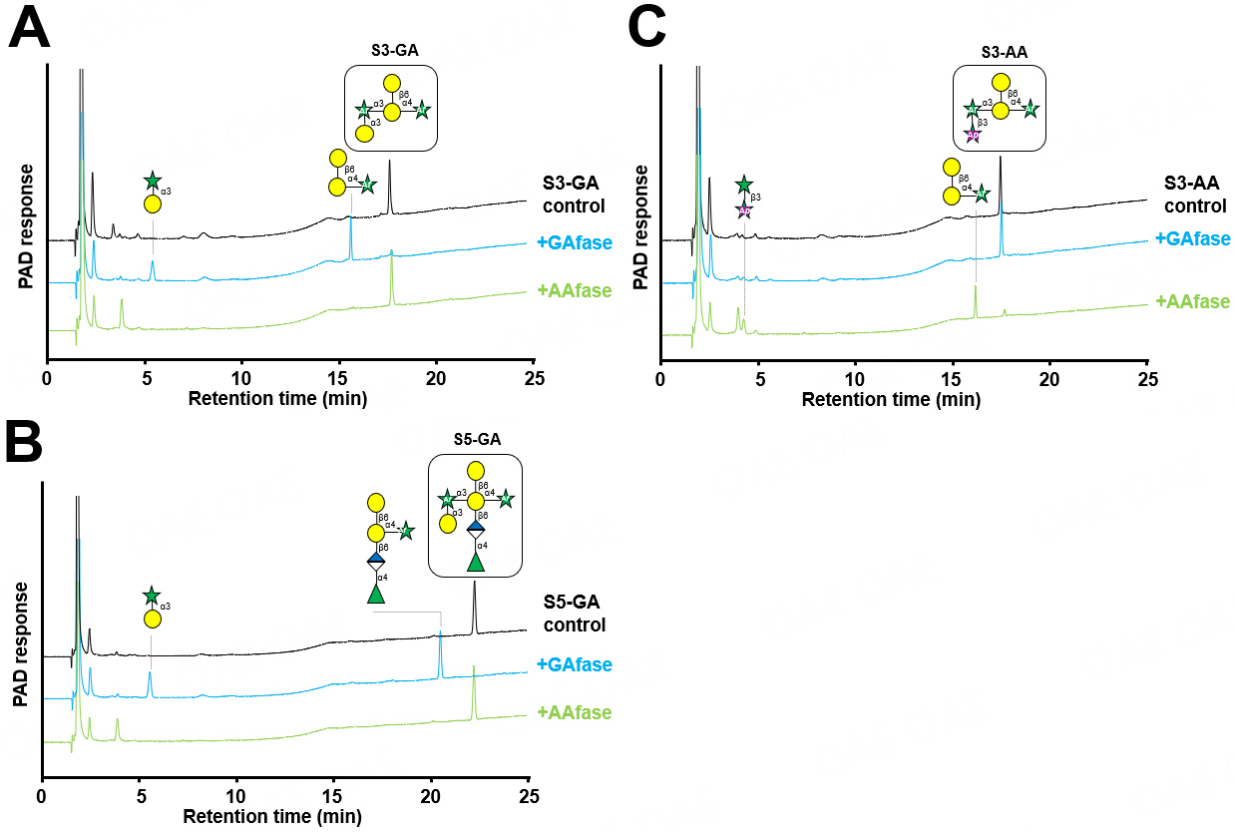
HPAEC-PAD analysis of AAfase reaction with oligosaccharides derived from gum arabic. AGP. HPAEC-PAD analysis of the reaction with gum arabic-related oligosaccharides. S3-GA (A), S3-AA (B), and S5-GA (C) were incubated with GAfase or AAfase at 37 °C for 24 h.

Next, experiments using oligosaccharides derived from gum arabic AGP (S3-GA, S3-AA, and S5-GA) as substrates were performed [Figure 3]. Under enzyme-limiting conditions, GAfase released α-D-Gal*p*-(1→3)-L-Ara from S3-GA and S5-GA, which corroborated with our previous study results; however, AAfase did not release this disaccharide. AAfase can act on S3-AA and release β-L-Ara*p*-(1→3)-L-Ara. Moreover, AAfase preferentially released β-L-Ara*p*-(1→3)-L-Ara over α-D-Gal*p*-(1→3)-L-Ara and thus had a different substrate specificity compared with GAfase. Based on substrate specificity, this enzyme was named 3-*O*-β-L-arabinopyranosyl-α-L-arabinofuranosidase. The *K*_m_, *k*_cat_, and *k*_cat_*/K*_m_ values of AAfase for larch AGP were 4.22 mg/mL, 63.2 ± 5.2 s^-1^, and 15.5 mL/mg/s, respectively. The optimal pH and temperature for AAfase activity were 6.5 and 35 °C-45 °C, respectively [Supplementary Figure 2].

### Amino acid residues involved in the substrate specificity of AAfase

**Figure 4:**
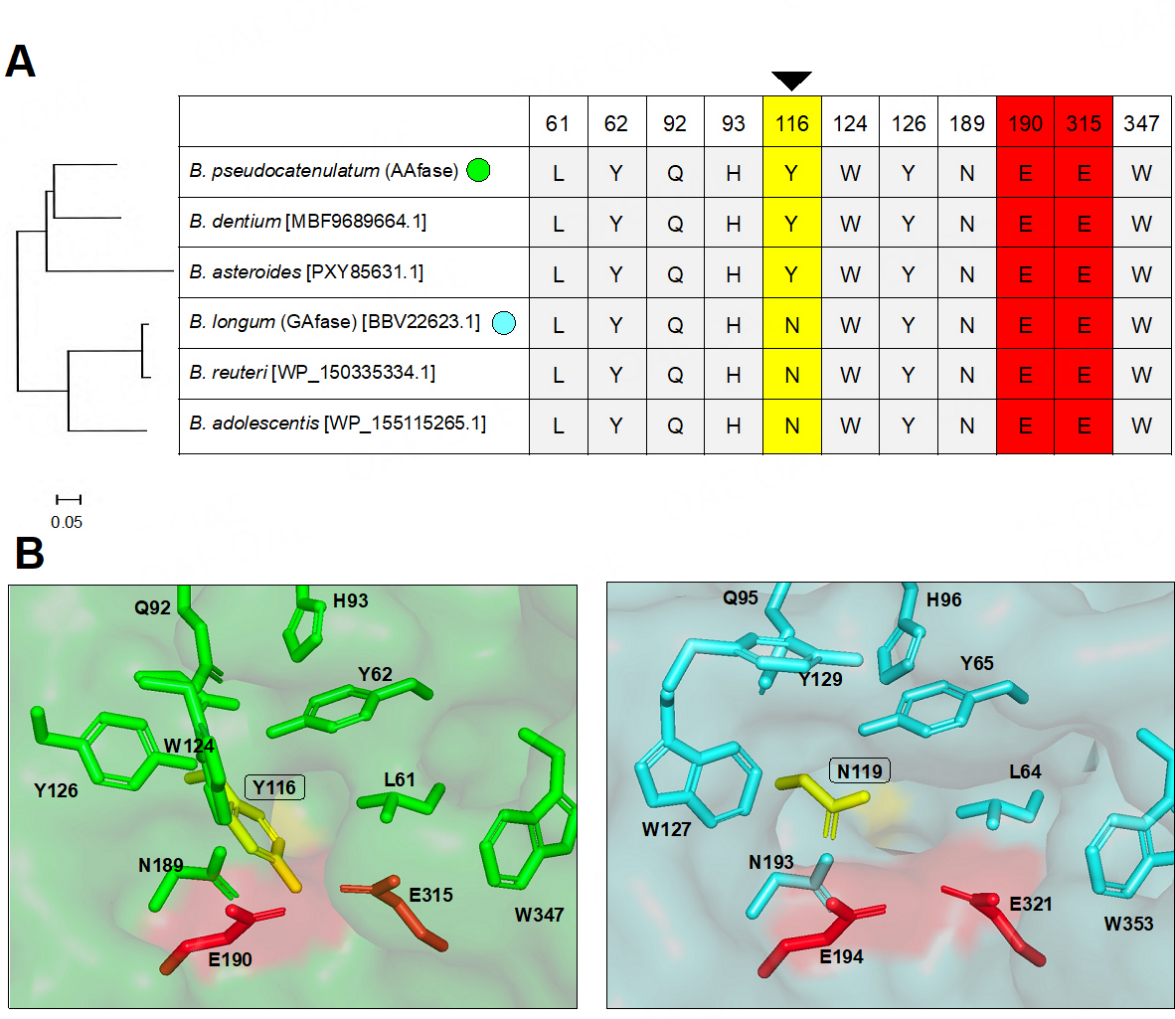
Comparison of amino acid residues at the catalytic site among Bifidobacterium GH39s. A: Comparison of amino acid residues at the catalytic sites, according to a previous report^[24]^. Catalytic residues (red) and residues with variation (yellow) across GH39 sequences are presented. The phylogenetic tree depicted on the left was constructed via the neighbor-joining method based on aligned Bifidobacterium GH39 sequences. GH39 sequences were aligned using the Clustal W program via MEGA11 software; B: The predicted structures of AAfase (left) and GAfase (right) are depicted as a surface representation. AlphaFold2 (ColabFold) was used to obtain the structural model with default settings. Predicted catalytic residues are highlighted in red, and different amino acid residues (Tyr^116^ in AAfase and Asn^119^ in GAfase) are highlighted in yellow.

AAfase and GAfase mainly released β-L-Ara*p*-(1→3)-L-Ara and α-D-Gal*p*-(1→3)-L-Ara from AGPs, respectively. To identify the amino acids that govern the differentiation of substrate specificity between AAfase and GAfase, the amino acids involved in the catalytic reaction were selected and compared among the bifidobacterial GH39 candidates based on the amino acid sequence of GH39 α-L-(β-1,2)-arabinofuranobiosidase (NF2152), whose crystal structure had been previously determined^[24]^ [Figure 4A, Supplementary Figure 3]. Amino acid sequences with 57%-75% identity with AAfase from the following species were termed “Bifidobacterium GH39s” in this study: homologous genes from *B. longum* (60% identity), *B. adolescentis* (57% identity), *B. dentium* (75% identity), *B. asteroides* (65% identity), and *B. reuteri* (59% identity) [Figure 4A]. The alignment of amino acid sequences showed that critical residues, including the putative acid/base catalytic residue (Glu^190^ in AAfase) and nucleophile (Glu^315^ in AAfase), were conserved across all GH39s, except for Tyr^116^ in AAfase that only differed from Asn in GAfase. The sequences of GAfase-type (Asn) and AAfase-type (Tyr) Bifidobacterium GH39s were divided into different groups in the phylogenetic tree. Based on the previously reported docking model of NF2152 and α-L-(β1,2)-arabinobiose^[24]^, the residue Glu^110^ in NF2152 is a counterpart of Asn^119^/Tyr^116^ in GAfase/AAfase and is located in the vicinity of C-5 position of L-arabinofuranose at the -2 subsite. Due to the different linkage patterns (1,2/1,3) and cyclic structures (furanose/pyranose) of substrates between NF2152 and AAfase/GAfase, the nonreducing end of the substrate was not necessarily accommodated in the same orientation as previously reported. The amino acid residue was hypothesized to be related to substrate recognition by GAfase and AAfase. Based on the structural simulation model via AlphaFold2 (ColabFold), the targeted amino acid residues, Tyr^116^ (AAfase)/Asn^119^ (GAfase), were predicted to be located on the far side of the substrate pocket [Figure 4B]. However, other amino acid residues "Tyr^126^ (AAfase)/Tyr^129^ (GAfase) and Trp^124^ (AAfase)/Trp^127^ (GAfase)" had different orientations in GAfase and AAfase.

To confirm whether an amino acid residue is involved in the differentiation of substrate specificity, a mutation was introduced into GAfase. We obtained a GAfase N119Y mutant and compared the substrate specificity between the wild-type (WT) and mutant using transglycosylated products (β-L-Ara*p*-(1→3)-α-L-Ara*f*-OMe and α-D-Gal*p*-(1→3)-α-L-Ara*f*-OMe) as substrates [Table 2]. The β-L-Ara*p*-(1→3)-α-L-Ara-releasing activity of AAfase WT was 195-fold higher than its α-D-Gal*p*-(1→3)-α-L-Ara-releasing activity, but that of GAfase WT was 556-fold lower. The GAfase N119Y mutant had 21.7-fold higher activity against β-L-Ara*p*-(1→3)-α-L-Ara*f*-OMe than against α-D-Gal*p*-(1→3)-α-L-Ara*f*-OMe, i.e., it converted “GAfase-type” activity into “AAfase-type” activity. Thus, Asn^119^ in GAfase and Tyr^116^ in AAfase are critical for determining substrate specificity. Moreover, the activity of another type of GAfase mutant, N119D, was measured using pre-desalting protein because the activity was lost by desalting. The substrate preference of the GAfase N119D mutant did not shift, which demonstrated its higher activity against α-D-Gal*p*-(1→3)-α-L-Ara*f*-OMe than against β-L-Ara*p*-(1→3)-α-L-Ara*f*-OMe, suggesting that the mutation to tyrosine is meaningful [Supplementary Figure 4].

**Table 2:** Substrate specificities of AAfase, GAfase, and GAfase N119Y mutant

**Substrate**	**AAfase** **(U/mg)**	**GAfase** **(U/mg)**	**GAfase N119Y** **(U/mg)**
α-D-Gal*p*-(1→3)-α-L-Ara*f*-OMe	0.0776	33.1	0.363
β-L-Ara*p*-(1→3)-α-L-Ara*f*-OMe	15.1	0.0595	7.87

### *In vitro* assimilation test of β-L-Ara*p*-(1→3)-L-Ara and larch AGP in *B. pseudocatenulatum*

**Figure 5:**
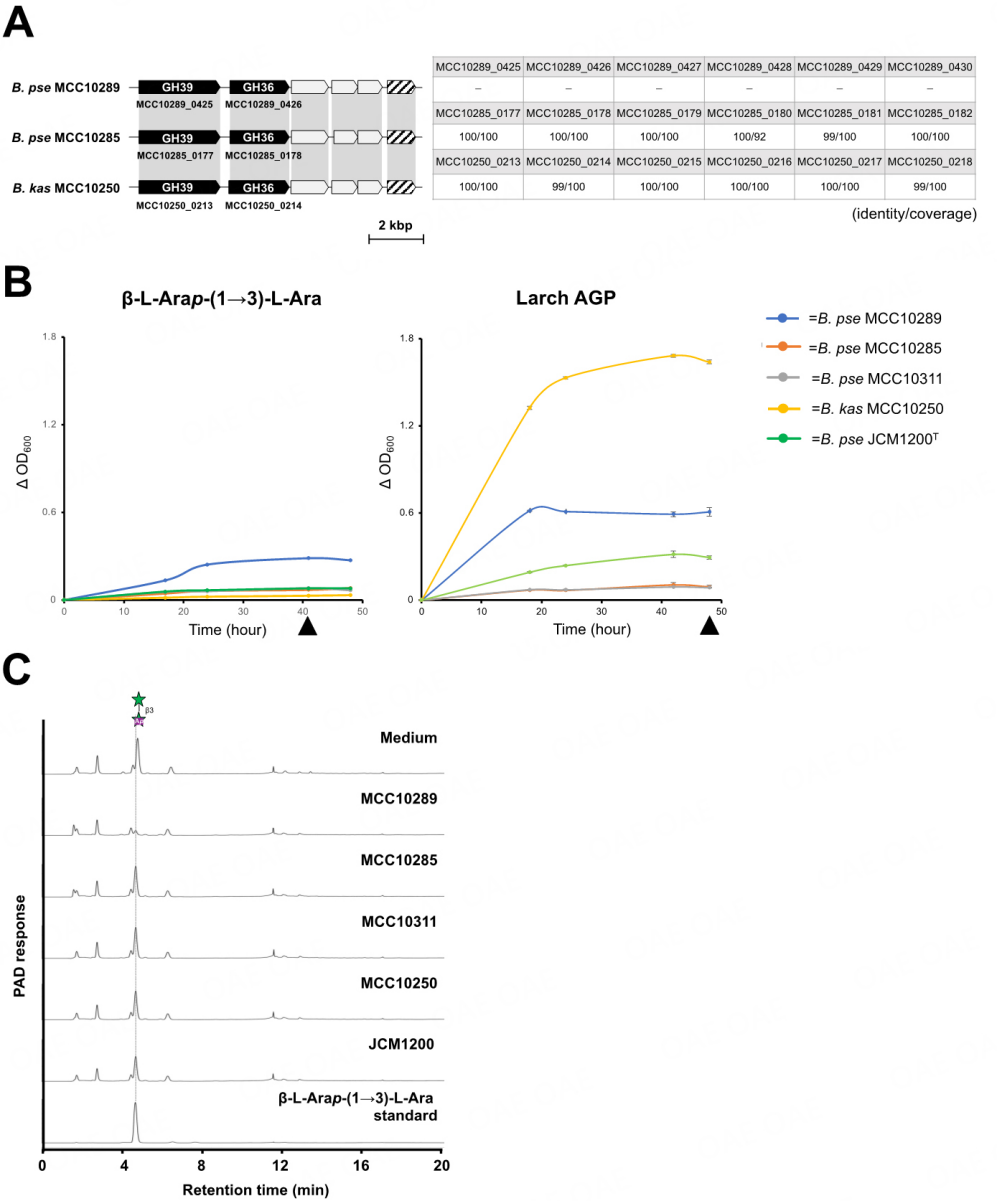
*In vitro* assimilation test of β-L-Ara*p*-(1→3)-L-Ara and larch AGP. *Bifidobacterium pseudocatenulatum* (*B. pse*) and *B. kashiwanohense* (*B. kas*) strains were used for the *in vitro* assimilation test. A: Gene clusters containing AAfase in *Bifidobacterium* species used in the growth test (left). Amino acid sequence identity and coverage between *B. pseudocatenulatum* MCC10289 and other strains using sequences from *B. pseudocatenulatum* MCC10289 as a query sequence (right table); B: The growth profile of β-L-Ara*p*-(1→3)-L-Ara (left) (*n* = 1) and larch AGP (right) (*n* = 3). The absorbance of media of β-L-Ara*p*-(1→3)-L-Ara was monitored at 0, 17, 24, 41, and 48 h, and that of larch AGP was monitored at 0, 18, 24, 42, and 48 h. The absorbance of the growth medium was calculated by subtracting the absorbance of the medium in the absence of bacteria. Error bars indicate standard deviation (*n* = 3). The black triangle indicates the sample collection point for residual sugar analysis via HPAEC-PAD or TLC; C: HPAEC-PAD analysis of the culture supernatant of β-L-Ara*p*-(1→3)-L-Ara after incubation for 41 h. β-L-Ara*p*-(1→3)-L-Ara was used as a standard.

AAfase mainly releases β-L-Ara*p*-(1→3)-L-Ara from gum arabic and larch AGP. The AAfase gene is flanked by genes encoding putative β-L-arabinopyranosidase and ABC transporter, and this gene cluster was expected to be involved in assimilating β-L-Ara*p*-(1→3)-L-Ara as a sugar source. Therefore, we performed the *in vitro* assimilation test of β-L-Ara*p*-(1→3)-L-Ara and larch AGP using AAfase-carrier (* B. pseudocatenulatum* MCC10289, *B. pseudocatenulatum* MCC10285, and *B. kashiwanohense* MCC10250) and noncarrier (*B. pseudocatenulatum* MCC10311 and *B. pseudocatenulatum* JCM1200^T^) *Bifidobacterium* strains [Figure 5]. Among AAfase-carrier strains, proteins encoded in the AAfase gene cluster share over 99% of sequence identity with the corresponding proteins from *B. pseudocatenulatum* MCC10289 [Figure 5A, right table]. The *in vitro* assimilation test of β-L-Ara*p*-(1→3)-L-Ara and larch AGP was performed by measuring the increase in absorbance and conducting residual sugar analysis in the culture supernatant via TLC and HPAEC-PAD. As a result, only *B. pseudocatenulatum* MCC10289 showed growth [Figure 5B, left], and HPAEC-PAD analysis indicated that β-L-Ara*p*-(1→3)-L-Ara was completely utilized by *B. pseudocatenulatum* MCC10289 [Figure 5C]. Unexpectedly, other AAfase-carrier strains, *B. pseudocatenulatum* MCC10285 and *B. kashiwanohense* MCC10250 did not grow and utilize β-L-Ara*p*-(1→3)-L-Ara. The *in vitro* assimilation test of larch AGP showed that MCC10289 exhibited higher absorbance than *B. pseudocatenulatum* MCC10285, MCC10311, and JCM1200. As β-L-Ara*p*-(1→3)-L-Ara was present only in ~1.2% (w/w) of the total sugar content in larch AGP^[8]^, the difference was not so large because of the insufficient amount of β-L-Ara*p*-(1→3)-L-Ara. Interestingly, *B. kashiwanohense* MCC10250, which does not grow on β-L-Ara*p*-(1→3)-L-Ara, showed good growth on larch AGP [Figure 5B], and some released oligosaccharides were detected in the culture medium after 48 h via TLC [Supplementary Figure 5].

To examine bacterial AAfase activity, the precipitates of bacterial cells and supernatant of the culture medium containing larch AGP were collected and incubated with larch AGP [Supplementary Figure 6]. TLC analysis showed bacterial AAfase activity in the supernatant of *B. pseudocatenulatum* MCC10289 but not in the precipitate, indicating that AAfase is a secreted enzyme. This finding is reasonable based on the predicted domain structure of the enzyme with the SP sequence but no transmembrane domain [Figure 1B]. No enzyme activities were detected using other AAfase-carrier strain bacterial cells or supernatant.

## DISCUSSION

This study characterized MCC10289_0425, a GH39 AAfase from *B. pseudocatenulatum* exhibiting β-L-Ara*p*-(1→3)-L-Ara-releasing activity against gum arabic, larch AGP, and sugar beet arabinan. An amino acid sequence comparison between AAfase and GAfase revealed that almost all amino acid residues involved in catalysis and sugar-binding were conserved. However, only Asn^119^, which is located at the -2 subsite, was replaced by tyrosine (Tyr^116^) in AAfase. Furthermore, we converted GAfase-like activity to AAfase-like activity by introducing a single amino acid substitution of Asn^119^ to Tyr. In a study by Ichinose *et al*., GH27 β-L-arabinopyranosidase from *Streptomyces avermitilis* (SaArap27A) was converted into α-galactosidase by substituting Asn^99^ with Glu^[25]^. Structural analysis of the SaArap27A-saccharide complex revealed that the targeted site, Glu^99^, is located near the O^6^ atom of 5-(hydroxymethyl) group of galactose-a group lacking the L-arabinopyranose molecule. α-D-Gal forms a strong hydrogen bond with the Glu^99^-O^ϵ1^ atom and reduces catalytic turnover, suggesting that Glu^99^ is suitable for β-L-arabinopyranosidase activity. In this study, Asn^119^ in GAfase and Tyr^116^ in AAfase recognized the nonreducing ends of α-galactopyranosyl and β-L-arabinopyranosyl disaccharide, respectively. The homologous genes with > 57% amino acid sequence identity with AAfase were categorized as Asn- and Tyr-type, exhibiting GAfase-like and AAfase-like activities, respectively. Depending on the species, these distinct substrate specificities would provide different fitness abilities to respond to diverse plant polysaccharides in the human gut environment. Tyr-type AAfase is a strain-specific enzyme with a prevalence of 36% in *B. pseudocatenulatum* (*n* = 168; identity > 97%, coverage > 99%), 39% in *B. dentium* (*n* = 47; identity > 73%, coverage > 99%), and 7.5% in *B. asteroides* (*n* = 40; identity > 63%, coverage > 95%) strains, according to the National Center for Biotechnology Information database. In contrast, Asn-type GAfase has a low prevalence in *B. longum* (6.8%, *n* = 307) and *B. adolescentis* (7.0%, *n* = 57) strains. Although the distribution of α-D-Gal-(1→3)-α-L-Ara*f*- and β-L-Ara*p*-(1→3)-α-L-Ara*f*- structures is not common to all AGPs, it may be present in other plant polysaccharides and AGPs in addition to gum arabic and larch AGP. Wheat AGP was reported to possess β-L-Ara*p*-(1→3)-α-L-Ara*f*- structure at the end of the side chain^[26]^. However, AAfase exhibited no such activity in this study, suggesting that AAfase requires other enzymes for trimming the sugars attached to the side chain of wheat AGP. Using mild hydrolysis, β-L-Ara*p*-(1→3)-L-Ara was detected in the soluble fractions of green seaweed (*Codium fragile*)^[27]^, and the terminal β-L-Ara*p*- structure was found in pectic arabinan in the roots of marshmallow (*Althaea officinalis*)^[28]^ and pigeon pea (*Cajanus cajan*)^[29]^. Thus, there may be other candidate substrates for AAfase.

*B. pseudocatenulatum* is found in human intestines from infants to adults. It was reported to possess the transport system for using human milk oligosaccharides^[30]^ and some GHs that act on plant polysaccharides, such as xylan^[10]^. We revealed that the GH39 AAfase acts on larch and gum arabic AGP as a strain-specific enzyme in *B. pseudocatenulatum*. This finding supports the hypothesis that *B. pseudocatenulatum* can survive in the human intestine owing to the presence of various plant polysaccharide-degrading enzymes. *In vitro* assimilation test revealed that *B. pseudocatenulatum* MCC10289 utilized β-L-Ara*p*-(1→3)-L-Ara as a sugar source. These results support the hypothesis that *B. pseudocatenulatum* MCC10289 can transport β-L-Ara*p*-(1→3)-L-Ara released by AAfase into the bacterial cell via an ABC transporter (MCC10289_0427-0429) and that β-L-Ara*p*-(1→3)-L-Ara is degraded into L-arabinose by the intracellular β-L-arabinopyranosidase (MCC10289_0426) and is metabolized as an energy source. However, other AAfase-carrier *Bifidobacterium* strains could not grow even though they possessed this gene cluster for assimilating β-L-Ara*p*-(1→3)-L-Ara. The amino acid sequences of the AAfase gene clusters within the AAfase-carrier strains are almost identical but have slight differences in the ABC transporter and intracellular GH36 [Figure 5A]. Although it is possible that the difference may affect the protein conformations, the relationship for the assimilation remains unclear. Interestingly, *B. kashiwanohense* MCC10250 showed good growth on larch AGP even though it does not encode the genes for type II AG degradative enzymes, such as main chain degrading enzyme (Bl1,3Gal)^[31]^, side chain degrading enzyme (Bl1,6Gal), and α-L-arabinofuranosidase (BlArafA)^[7]^, which had been characterized in *B. longum* JCM1217. Therefore, this strain probably has other uncharacterized enzymes that degrade β-1,3/1,6-galactan and/or α-L-arabinofuranoside chains on larch AGP.

GH39 GAfase homologs are widely conserved across *Bifidobacterium* strains and are novel candidates for AGP-degrading enzymes. The GH39 homolog from *B. catenulatum* JCM1194, which has 28% amino acid sequence identity with GAfase, is adjacent to the GH127 putative β-L-arabinofuranosidase gene. Considering that the neighboring gene tends to be associated with a series of reactions, it may degrade and release an oligosaccharide containing β-L-Ara*f* structures. The side chain structure of AGP varies depending on plant species, *e.g.*, the side chain structure of gum arabic AGP from *Acacia seyal* differs from that of gum arabic AGP from *Acacia senegal*, in which it is substituted by L-Ara*f* oligomers instead of α-D-Gal-(1→3)-α-L-Ara*f*-(1→3)^[32]^. Further investigations are warranted to elucidate the functional diversity of *Bifidobacterium* GH39s corresponding to the structural diversity of AGPs.

## DECLARATIONS

### Availability of data and materials

The sequence of the synthesized *MCC10289_0425* gene has been deposited in the DDBJ databank under the accession number LC745705.

### Copyright

© The Author(s) 2023.
