# Supplementary material for: Assimilation of arabinogalactan side chains with novel 3-O-β-L-arabinopyranosyl-α-L-arabinofuranosidase in Bifidobacterium pseudocatenulatum
Source: Microbiome Res Rep. 2023 Apr 15;2(2):12. doi: 10.20517/mrr.2023.08 (PMC10688797; doi:10.20517/mrr.2023.08)
Supplement: Supplementary file 1 [file mrr-2-2-12-SupplementaryMaterials.pdf]

Microbiome Research Reports

Supplementary Material

Different fitness to arabinogalactan side chains is supported by diversified specificity of glycoside hydrolase family 39 enzymes in the genus *Bifidobacterium*

Yuki Sasaki[1,2], Makoto Yanagita[1], Mimika Hashiguchi[1], Ayako Horigome[3], Jin-Zhong Xiao[3], Toshitaka Odamaki[3], Kanefumi Kitahara[1], Kiyotaka Fujita[1]

[1]Faculty of Agriculture, Kagoshima University, Kagoshima, Kagoshima 890-0065, Japan.

[2]Graduate School of Biostudies, Kyoto University. Sakyo-ku, Kyoto 606-8502, Japan.

[3]Next Generation Science Institute, Morinaga Milk Industry Co. Ltd., Zama, Kanagawa 252-8583, Japan.

Correspondence to: Prof. Kiyotaka Fujita. Faculty of Agriculture, Kagoshima University, 1-21-24 Korimoto, Kagoshima 890-0065, Japan. E-mail: k4022897@kadai.jp

© The Author(s) 2021. Open Access This article is licensed under a Creative Commons Attribution 4.0 International License (https://creativecommons.org/licenses/by/4.0/), which permits unrestricted use, sharing, adaptation, distribution and reproduction in any medium or format, for any purpose, even commercially, as long as you give appropriate credit to the original author(s) and the source, provide a link to the Creative Commons license, and indicate if changes were made.

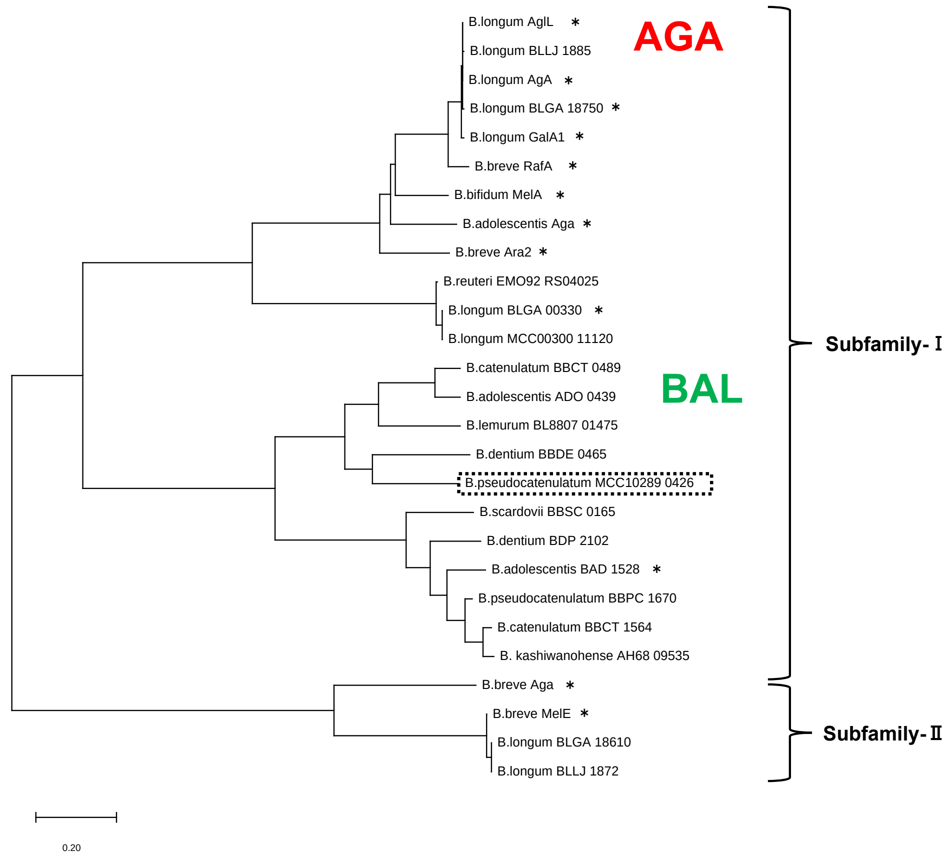

Supplementary Figure 1. The phylogenetic relationships of bifidobacterial GH36 α-galactosidases and β-L-arabinopyranosidases. The phylogenetic tree of *B. pseudocatenulatum* MCC10289_0426 with homologous proteins from bifidobacteria was constructed by the neighbor-joining method using the aligned sequences; for the construction, the program Clustal W was implemented in the MEGA11 software. *B. pseudocatenulatum*_MCC10289_0426 is indicated by a dashed-line box. The protein names or locus tags are shown alongside *Bifidobacterium* strains as follows: *B. adolescentis* Aga (GenBank ID: AAD30994.2), *B. bifidum* MelA (ABD96085.1), *B. breve* Aga (AAK96217.2), *B. breve* Aga2 (ABB76662.1), *B. longum* GalA1 (ACD98928.1), *B. longum* AgA (AAN25312.1), *B. longum* AglL (AAG02023.1), *B. breve* RafA (ABE96531.1), *B. breve* MelE (ABE96518.1), *B. longum* BLLJ_1872 (BAJ67536.1), *B. longum* BLLJ_1885 (BAJ67549.1), *B. longum* BlAga3 (BBV22622.1), *B. longum* BlAga1 (BBV24464.1), and *B. longum* BlAga2 (BBV24450.1), *B. longum* MCC00300_11120 (WP_077381863.1), *B. reuteri* EMO92_RS04025 (WP_150335335.1), *B. catenulatum* BBCT_0489 (BAR01457.1), *B. adolescentis* BADO_0439 (AII75864.1), *B. lemurum* BL8807_01475

(QOL34630.1), *B. dentium* BBDE_0465 (BAQ26459.1), *B. adolescentis* BAD_1528 (BBD14080.1), *B. pseudocatenulatum* BBPC_1670 (BAR04348.1), *B. catenulatum* BBCT_1564 (BAR02532.1), *B. kashiwanohense* AH68_09535 (AIZ15229.1), *B. dentium* BDP_2102 (ADB10663.1), and *B. scardovii* BBSC_0165 (BAQ30245.1). Asterisk indicates characterized enzymes.

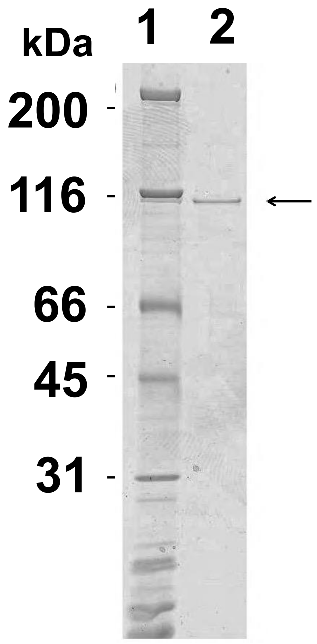

Supplementary Figure 2. SDS-PAGE analysis of recombinant AAfase. Purified AAfase was electrophoresed on a 5–20% gradient polyacrylamide gel and stained with Coomassie Brilliant Blue R-250. Lane 1, molecular size marker; lane 2, purified AAfase. Arrow indicates target protein at expected molecular size.

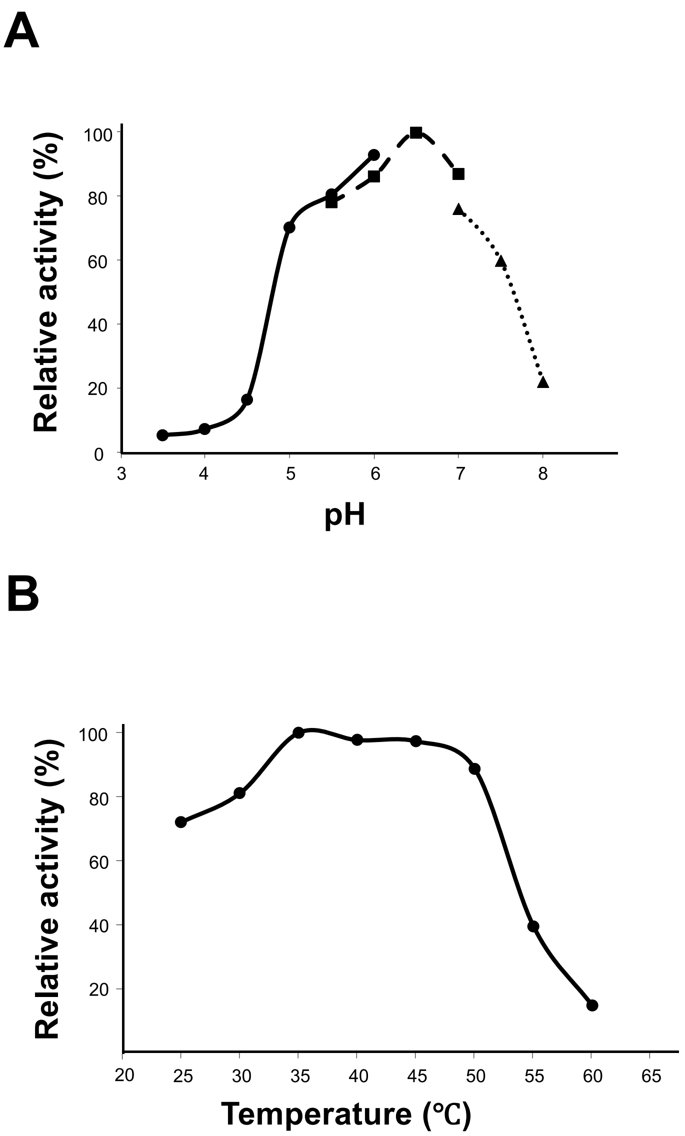

Supplementary Figure 3. Optimal pH and temperature of AAfase. (A) pH dependence of AAfase activity in various buffers at 45 °C for 20 min. Sodium acetate buffer (closed circle and solid line), MES buffer (closed square and dashed line), and HEPES buffer (closed triangle and dotted line) were used. Enzyme activities are expressed as a percentage of the activity in MES buffer at pH 6.5. (B) The temperature dependence of AAfase activity at pH 6.5 for 20 min. The enzymatic activities are expressed as the percentage of the activity at 35 °C.

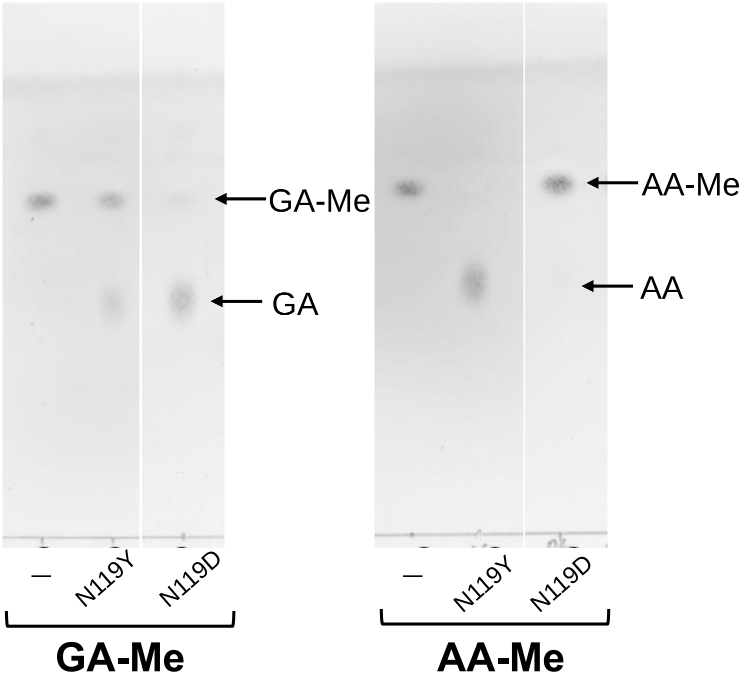

Supplementary Figure 4. TLC analysis of the enzymatic activity of GAfase N119Y and GAfase N119D. GA-Me and AA-Me are the abbreviation of α-D-Gal-(1→3)-α-L-Ara*f*-OMe and β-L-Ara*p*-(1→3)-α-L-Ara*f*-OMe, respectively. 1 mM GA-Me and AA-Me were incubated with absence (−) or presence (＋) of the two types of mutants in 50 mM sodium acetate buffer (pH 5.0) at 37 °C for 18 h. As enzymes, the elution fraction containing imidazole after His-tag affinity purification were used in this study, because desalted GAfase N119D got lost enzymatic activity. The amount of enzyme was matched when the same enzyme was used in order to see the activity on different substrates, although the quantity of N119Y and N119D were different. The reaction mixtures were analyzed by TLC, as described in main text.

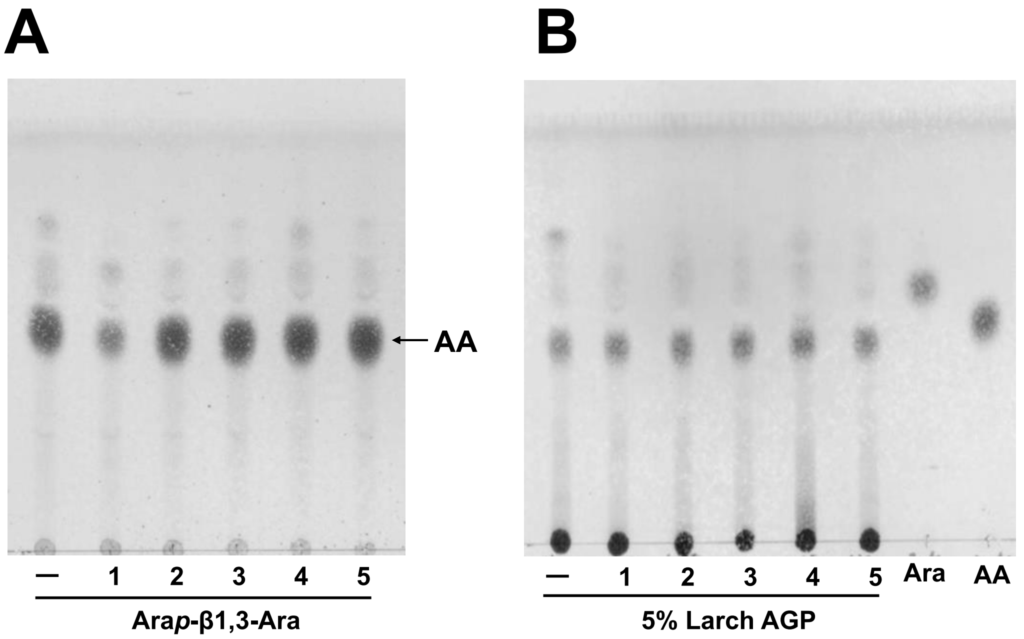

Supplementary Figure 5. TLC analysis of the culture supernatants after incubation for 41 hours in β-L-Ara*p*-(1→3)-L-Ara (A) and and 48 hours in larch AGP (B). 1, *B. pseudocatenulatum* MCC10289; 2, *B. pseudocatenulatum* MCC10285; 3, *B. pseudocatenulatum* MCC10311; 4, *B. kashiwanohense* MCC10250; 5, *B. pseudocatenulatum* JCM1200^{T}. As a control, the medium without any bacteria (−) was used. AA is the abbreviation for β-L-Ara*p*-(1→3)-L-Ara.

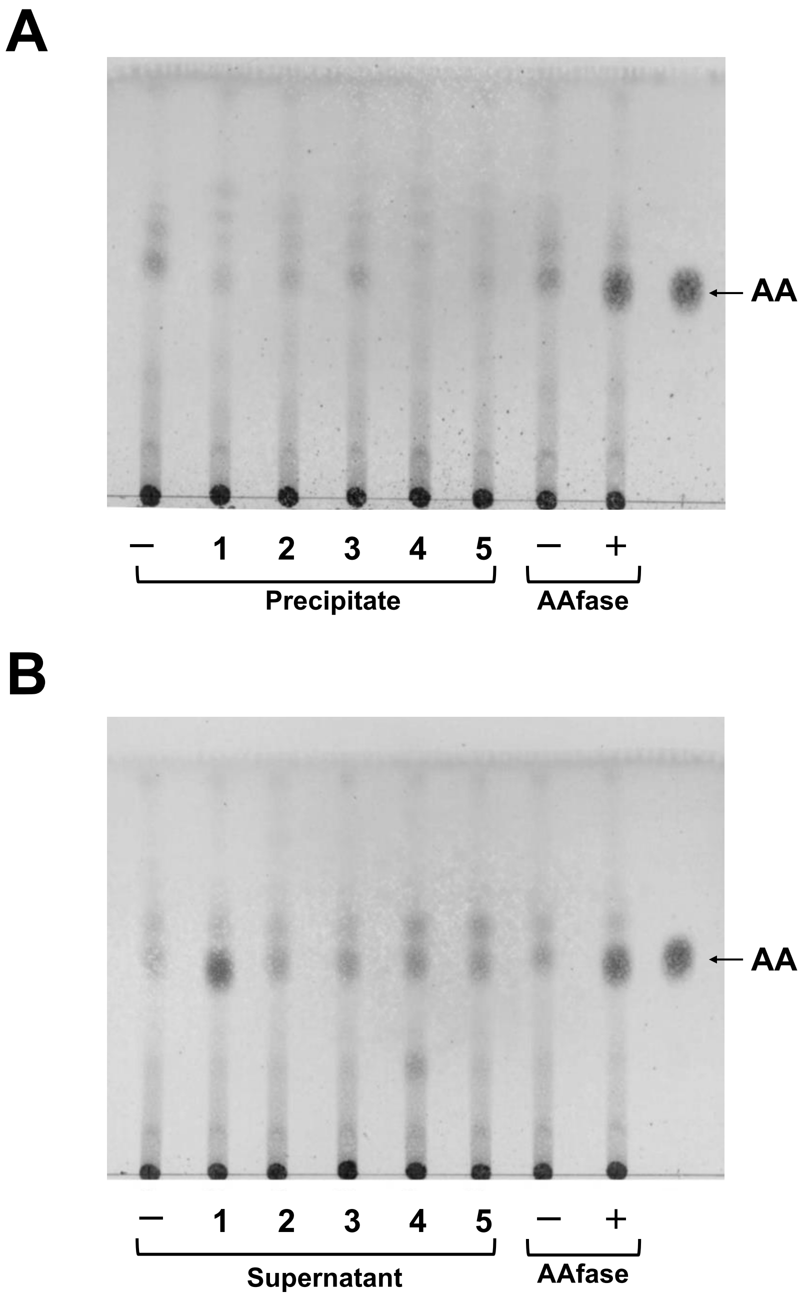

Supplementary Figure 6. TLC analysis of the bacterial enzymatic activities with 2.5% larch AGP. Larch AGP was incubated with bacterial cell fractions (A) and culture supernatant (B) after cultivation of B. pseudocatenulatum MCC10289 (1), B. pseudocatenulatum MCC10285 (2), B. pseudocatenulatum MCC10311 (3), B. kashiwanohense MCC10250 (4), and B. pseudocatenulatum JCM1200T (5) grown in larch AGP after 18 h incubation. As a control, the medium without any bacteria (−) was used for enzymatic activity assay. As a control, larch AGP was incubated in the

absence (−) or presence (+) of AAfase.
